# Nutritional remission without mucosal healing in type II refractory celiac disease: a case report

**DOI:** 10.3389/fnut.2026.1941830

**Published:** 2026-08-28

**Authors:** Om Kolthoum Sallem, Asma Sabbek, Nabil Ben Chaabène, Leila Safer

**Affiliations:** 1 Department of Clinical Nutrition, Fattouma Bourguiba University Hospital, Faculty of Medicine of Monastir, University of Monastir, Monastir, Tunisia; 2 Department of Gastroenterology, Fattouma Bourguiba University Hospital, Faculty of Medicine of Monastir, University of Monastir, Monastir, Tunisia

**Keywords:** budesonide, gluten-free diet, malnutrition, Mediterranean, nutritional rehabilitation, parenteral nutrition, refractory celiac disease, Tunisia

## Abstract

Type II refractory celiac disease (RCD II) is a rare pre-lymphomatous complication of celiac disease associated with severe malabsorption and a high risk of enteropathy-associated T-cell lymphoma. We report a 56-year-old Tunisian woman with adult-onset celiac disease who developed progressive diarrhea and life-threatening malnutrition despite dietitian-verified adherence to a gluten-free diet. At admission, she weighed 34 kg (body mass index, 12.9 kg/m^2^), was hypotensive and wheelchair dependent, and reported up to 20 stools/day. She had anemia, hypoalbuminemia, and hypophosphatemia. Computed tomography enterography, whole-body 18F-FDG PET/CT, jejunoscopy, and ileocolonoscopy found no lymphoma. Duodenal biopsies showed total villous atrophy and >50% aberrant CD3+/CD7+/CD8−/CD30− intraepithelial lymphocytes, supporting RCD II; flow cytometry and T-cell receptor clonality testing were unavailable. Treatment comprised 1 month of exclusive parenteral nutrition with refeeding-syndrome prophylaxis, 1 month of combined enteral and oral feeding, and subsequent oral nutrition. After systemic corticosteroids failed, open-capsule budesonide was initiated at 6 mg/day and reduced to 3 mg/day after 2 months. At 4 months, villous atrophy and the aberrant lymphocyte population were unchanged. At 6 months, however, the patient had gained 26 kg, stool frequency had fallen to 2-3/day, albumin had normalized, and mobility had improved. This case illustrates that nutritional recovery and intestinal disease activity may diverge in RCD II. Clinical and nutritional remission should not replace endoscopic and immunophenotypic surveillance.

## 1 Introduction

Celiac disease affects approximately 0.7% of the global population. In Tunisia, school-based screening has documented a prevalence of approximately 1 in 157 children, confirming that the disease is established across the southern Mediterranean (1, 2). Across the Mediterranean region, the clinical spectrum has shifted from a predominantly pediatric malabsorptive syndrome toward diagnosis throughout adulthood, including subtle and atypical presentations (3). This changing spectrum has implications for recognition of complicated disease phenotypes.

Refractory celiac disease (RCD) is defined by persistent or recurrent symptoms and villous atrophy despite at least 12 months of a strict gluten-free diet (GFD), after ongoing gluten exposure and alternative diagnoses have been excluded (4–6). RCD II is characterized by a clonal or aberrant intraepithelial lymphocyte (IEL) population and is increasingly regarded as a pre-lymphomatous, low-grade intraepithelial T-cell neoplasm (5–8). Diagnostic thresholds depend on the technique used: >50% aberrant IELs by immunohistochemistry or approximately 20–25% by flow cytometry supports RCD II (5, 8). Prognosis is poor because of severe malnutrition and the risk of progression to enteropathy-associated T-cell lymphoma (EATL) (9–12).

Published reports generally describe nutrition as supportive care, while paired nutritional, histological, and immunophenotypic outcomes are reported less often. We describe a Tunisian patient with extreme malnutrition who achieved complete weight restoration and broad biochemical recovery despite persistent villous atrophy and an unchanged aberrant IEL population. The case highlights both the life-saving role of structured nutritional rehabilitation and the need to evaluate nutritional recovery separately from intestinal disease activity. This case report was prepared in accordance with the CARE reporting guideline (25).

## 2 Case description

### 2.1 Patient information and previous interventions

A 56-year-old Tunisian woman was admitted in September 2022 with chronic diarrhea and severe malnutrition. Her medical history included chronic hepatitis B treated with entecavir since 2012; mycosis fungoides treated with phototherapy and retinoid analogs since 2013; and non-COVID-19 hypoxemic pneumonia in January 2022 complicated by hemophagocytic lymphohistiocytosis. The available record contained no contributory family history. HLA-DQ2/DQ8 typing and other germline genetic testing were not available. No formal psychosocial assessment was documented; the functional and emotional effects of progressive dependence are reported in the Patient Perspective section.

Celiac disease had been diagnosed in 2018 after chronic diarrhea and biochemical evidence of malabsorption. Anti-endomysial antibodies were positive, IgA anti-tissue transglutaminase was strongly positive (titer 1:200), and duodenal biopsies showed total villous atrophy with intraepithelial lymphocytosis (Marsh IIIc). A strict GFD was prescribed. Persistent diarrhea prompted reassessment in 2019: cross-sectional imaging and endoscopy excluded lymphoma, while lymphocytic colitis was diagnosed and treated with budesonide, with only partial improvement. During the next 3 years, diarrhea persisted and nutritional status deteriorated. In the 3 months before admission, stool frequency increased to 20/24 h. Detailed reassessment by an experienced dietitian confirmed satisfactory adherence to the GFD, and celiac serology had become negative. This verification was important because inadvertent gluten exposure is the most common cause of non-responsive celiac disease (13).

### 2.2 Clinical findings

**Figure 1:**
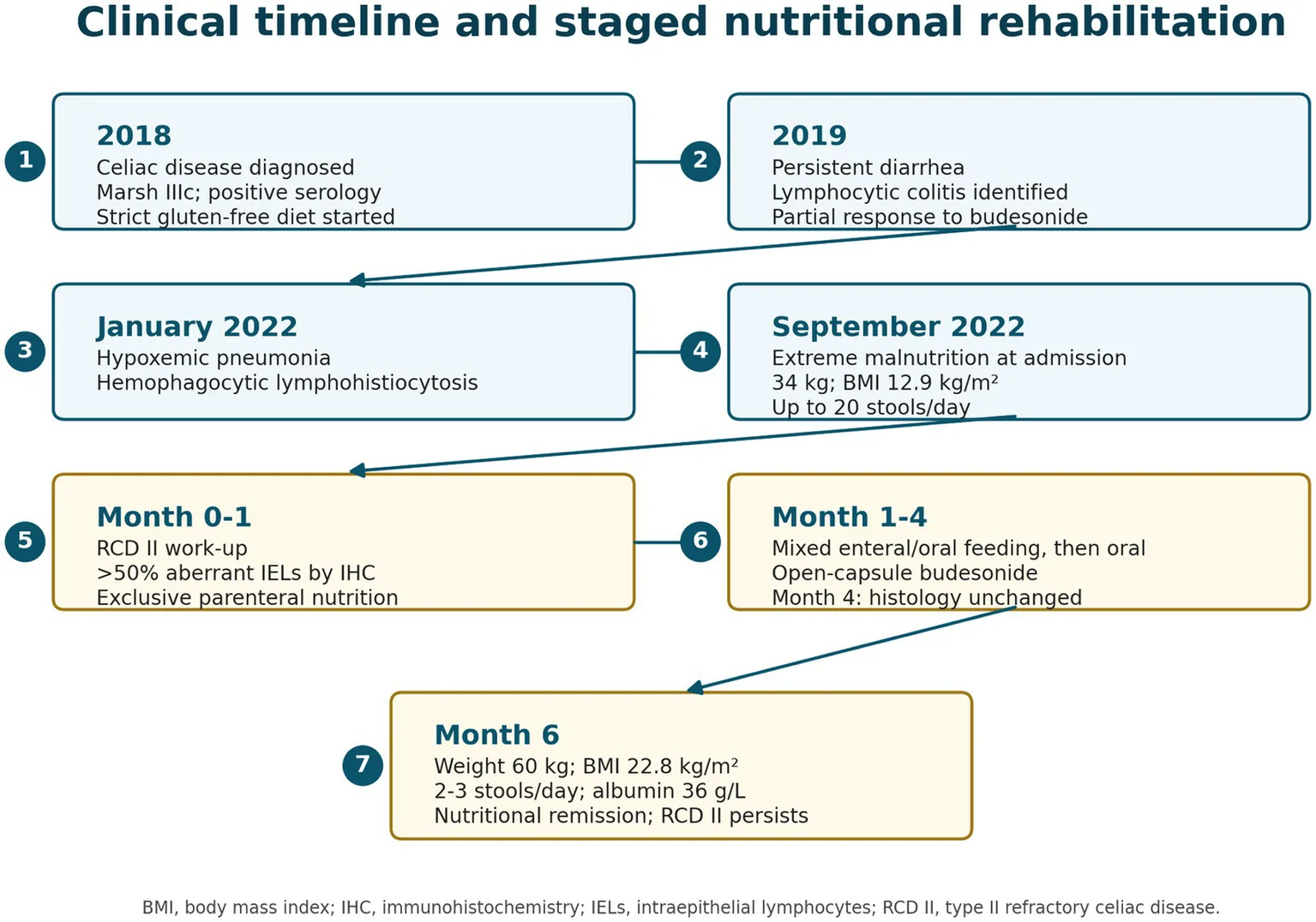
Timeline of the episode of care. The timeline summarizes the diagnosis of celiac disease in 2018, partial response after lymphocytic colitis was identified in 2019, severe deterioration by September 2022, RCD II diagnosis, staged nutritional rehabilitation, and the discordant 4- and 6-month outcomes.

At admission, the patient was profoundly asthenic, wheelchair dependent, and unable to maintain adequate intake. She weighed 34 kg, corresponding to a body mass index (BMI) of 12.9 kg/m^2^, and her mid-upper arm circumference was 13 cm. She had extracellular dehydration and hypotension (80/40 mmHg). Disseminated pruritic macules were consistent with known mycosis fungoides. No peripheral lymphadenopathy or hepatosplenomegaly was found. Table 1 summarizes the principal clinical, nutritional, and laboratory findings; Figure 1 presents the chronology of the episode of care.

**Table 1:** Exact paired clinical, biochemical, and histopathological findings at admission and follow-up.

Parameter	Admission	Follow-up	Interpretation
Body weight (kg)	34	60 (6 months)	+26 kg
Body mass index (kg/m^2^)	12.9	22.8 (6 months)	Restored to reference range
Stool frequency (per 24 h)	Up to 20	2–3 (6 months)	Marked clinical response
Hemoglobin (g/dL)	8.9	11.0 (6 months)	Improved; mild anemia persisted
Prothrombin time (%)	58	83 (6 months)	Improved to reference range
Serum albumin (g/L)	27	36 (6 months)	Normalized
Duodenal villous architecture	Total villous atrophy	Total villous atrophy (4 months)	No mucosal healing
Aberrant IEL population	>50%; CD3+/CD7+/CD8−/CD30−	>50%; CD3+/CD8− (4 months)	No immunophenotypic improvement

Only exact numerical or objectively documented paired findings available in the source case summary are shown. Normalization of mid-upper arm circumference, phosphate, sodium, urea, and vitamin B12 was documented, but the exact follow-up results could not be retrieved; these variables were therefore not presented as numerical outcomes. IEL, intraepithelial lymphocyte.

### 2.3 Diagnostic assessment

The immediate diagnostic priority was to exclude EATL and ulcerative jejunitis. Lactate dehydrogenase and beta-2-microglobulin were normal. Computed tomography enterography showed no bowel-wall thickening or lymphadenopathy, and whole-body 18F-FDG PET/CT showed no pathological uptake; these modalities are complementary in complicated or refractory celiac disease (14, 15). Upper endoscopy showed an atrophic, nodular duodenal mucosa. Jejunoscopy and ileocolonoscopy were macroscopically normal, with no ulcerative jejunitis. Video capsule endoscopy, which may provide additional information on distal small-bowel involvement, was unavailable (16).

Duodenal biopsies confirmed total villous atrophy, crypt hyperplasia, and marked intraepithelial lymphocytosis without overt lymphoma. Jejunal biopsies were normal. Immunohistochemistry showed that >50% of IELs expressed CD3 and CD7, lacked CD8, and were CD30 negative, exceeding the accepted immunohistochemical threshold for RCD II (5, 8, 17). Flow cytometry and T-cell receptor (TCR) clonality analysis, which are recommended complementary cornerstones for distinguishing RCD I from RCD II, were unavailable. The classification was therefore supported by the clinical course, expert histopathology, and the >50% aberrant IEL immunohistochemical phenotype, but lacked complete flow-cytometric and molecular confirmation. Skin biopsies excluded Sézary syndrome and progression of mycosis fungoides. Stool and biochemical investigations found no infection, exocrine pancreatic insufficiency, or thyroid dysfunction. The final working diagnosis was RCD II with extreme malnutrition and no demonstrable EATL. The differential diagnoses evaluated and the basis for their exclusion are summarized in Table 2.

**Table 2:** Differential diagnoses considered and their evaluation in this patient.

Differential diagnosis	Evaluation in this patient	Interpretation
Ongoing gluten exposure	Detailed review by an experienced dietitian; celiac serology became negative	Unlikely to explain persistent deterioration
EATL or ulcerative jejunitis	CT enterography, whole-body 18F-FDG PET/CT, jejunoscopy, ileocolonoscopy, and mucosal biopsies	No evidence at baseline work-up; surveillance remains necessary
Enteric infection	Stool investigations performed during reassessment	No infection identified
Exocrine pancreatic insufficiency	Stool and biochemical investigations	No evidence identified; exact assay results were not retained
Thyroid dysfunction	Biochemical thyroid assessment	No thyroid dysfunction identified
Lymphocytic colitis	Diagnosed in 2019; budesonide produced only partial improvement	Possible contributor, but insufficient to explain progressive malnutrition
Small-intestinal bacterial overgrowth	Clinically suspected and treated empirically with antibiotics	Not microbiologically confirmed; independent contribution cannot be determined

The table summarizes the differential diagnoses documented in the available source record and is not intended as an exhaustive differential.

### 2.4 Therapeutic intervention

Management addressed the intestinal disease and the immediate nutritional threat in parallel. Because severe diarrhea and malabsorption prevented adequate oral or enteral intake, exclusive parenteral nutrition was administered intravenously during month 1. Energy delivery was advanced cautiously because the BMI of 12.9 kg/m^2^ and phosphate concentration of 0.63 mmol/L indicated a very high risk of refeeding syndrome. Thiamine was administered and phosphate, magnesium, and potassium were monitored and replaced. Iron, vitamin B12, and fat-soluble vitamins were also repleted. During month 2, enteral nutrition and oral feeding were introduced progressively to a combined target of approximately 1,500 kcal/day. Exclusive oral nutrition was subsequently achieved as tolerance and voluntary intake improved. The source case summary did not retain the enteral access device or formula composition. The strict GFD was maintained throughout. A course of antibiotics was administered for suspected small-intestinal bacterial overgrowth; the agent, dose, route, and duration were not retained in the source case summary.

Systemic corticosteroid therapy at 1 mg/kg/day for 1 month produced no meaningful clinical or biochemical response and was tapered with hydrocortisone replacement. Open-capsule budesonide was then selected because the enteric formulations used in routine practice release drug predominantly in the distal small bowel and right colon, whereas celiac enteropathy is usually most marked proximally. Opening the capsule was intended to increase topical exposure of the proximal small-bowel mucosa. Budesonide was also favored over continued systemic corticosteroid exposure because of its extensive first-pass metabolism and the patient’s chronic hepatitis B, mycosis fungoides, and previous hemophagocytic lymphohistiocytosis (5, 18, 19).

The patient received budesonide orally at a total dose of 6 mg/day from opened capsules. The capsules were opened immediately before administration; whether the granules were ground or mixed with food was not documented. After 2 months, the dose was reduced to 3 mg/day following clinical stabilization and to limit cumulative corticosteroid exposure. This individualized regimen was lower than the commonly reported open-capsule protocol of 9 mg/day (3 mg three times daily) (5, 18, 19). No further intervention change was documented during the 6-month observation period. The GFD and oral nutritional rehabilitation were continued.

### 2.5 Follow-up and outcomes

Stool frequency decreased from up to 20 to 2–3/24 h, and abdominal pain resolved. Over 6 months, the patient gained 26 kg, reaching 60 kg and a BMI of 22.8 kg/m^2^. She regained the ability to walk and recovered partial autonomy. Serum albumin increased from 27 to 36 g/L, prothrombin time from 58 to 83%, and hemoglobin from 8.9 to 11.0 g/dL, although mild anemia persisted (Table 1). The source summary also documented normalization of phosphate, sodium, urea, vitamin B12, and mid-upper arm circumference, but the exact follow-up values were not retrievable and were therefore not reproduced in Table 1. No clinically recognized adverse event attributable to nutritional rehabilitation or budesonide was documented. Medication adherence was assessed clinically during follow-up; no validated adherence instrument or drug-level assessment was used.

Upper endoscopy 4 months after budesonide initiation showed persistent atrophic, nodular duodenal mucosa. Histology and immunohistochemistry were unchanged, with total villous atrophy and >50% aberrant CD3+/CD8− IELs. Budesonide was continued, and structured clinical, biochemical, endoscopic, and lymphoma surveillance was instituted. At 6 months, the patient remained in clinical and nutritional remission without evidence of lymphomatous transformation.

Six months was selected as the reporting endpoint because it was the longest interval for which complete paired clinical, nutritional, biochemical, and functional data were available after staged rehabilitation. It was not intended to define an evidence-based treatment duration or stopping rule. Published RCD studies used variable budesonide durations, and initial dose and tapering strategies have not been evaluated rigorously (5, 18, 20).

## 3 Discussion

The central observation is that clinical and nutritional recovery occurred without demonstrable mucosal or immunophenotypic improvement. The patient gained 26 kg, normalized most biochemical abnormalities, and recovered mobility, while total villous atrophy and the aberrant IEL population persisted. Weight, albumin, stool frequency, and functional status are valuable measures of recovery, but in RCD II they cannot be considered surrogate markers for control of the pre-lymphomatous intestinal lesion.

Direct comparison with previous budesonide studies places this discordance in context. In the closed-capsule series by Brar et al. (20), 29 patients received budesonide for a mean of 6.7 months; 76% achieved a clinical response, but no histological response was observed. This resembles the clinical-histological dissociation in our patient. By contrast, Mukewar et al. (18) treated 57 patients, including 13 with RCD II, with open-capsule budesonide: 92% improved clinically and 89% improved histologically; 7 of 13 patients with RCD II lost the previously detected clonal or aberrant IEL phenotype on follow-up biopsy. In the more recent cohort of Saitta et al. (19), 35 patients received open-capsule budesonide at 3 mg three times daily, while 16 received closed-capsule budesonide at 9 mg once daily. Open-capsule treatment was associated with greater mucosal healing (*p* < 0.001) and symptom improvement (*p* = 0.002), although only six patients had RCD II.

Our patient therefore differs from the majority in the open-capsule cohorts because the villous atrophy and aberrant IEL proportion were unchanged at 4 months despite marked clinical improvement. Several factors may explain this difference: RCD II is biologically less responsive than RCD I, the biopsy was obtained relatively early, the patient received 6 mg/day rather than the 9 mg/day protocol used in the principal open-capsule studies, and the exact manipulation of the capsule contents was incompletely documented. These differences preclude attributing the nutritional response to budesonide alone; staged nutrition, strict gluten exclusion, correction of deficiencies, and treatment of suspected bacterial overgrowth were concurrent interventions.

The 6-month observation period requires cautious interpretation. It corresponds to the longest complete paired follow-up in this case, not to a validated duration of therapy. Brar et al. reported a mean treatment duration of 6.7 months, whereas Mukewar et al. reported a median open-capsule duration of 15 months, with many patients remaining dependent on 3–6 mg/day maintenance therapy (18, 20). The AGA expert review notes that steroid starting doses and tapering strategies have not been studied rigorously (5). Consequently, the unchanged 4-month biopsy cannot establish permanent treatment failure. Because persistent aberrant IELs in RCD II confer an ongoing risk of progressive enteropathy, ulcerative jejunitis, and EATL, surveillance must extend well beyond 6 months. No validated surveillance interval exists; the planned strategy comprises regular multidisciplinary clinical and nutritional review, serial laboratory assessment, repeat endoscopy with biopsy, and small-bowel or cross-sectional imaging when clinically indicated (5, 6).

Nutritional rehabilitation was not ancillary treatment. A BMI of 12.9 kg/m^2^ accompanied by hypotension, hypoalbuminemia, and hypophosphatemia represented an immediate threat to survival. The route was selected according to intestinal tolerance and the ability to meet requirements: temporary parenteral nutrition was followed by graded enteral and oral reintroduction. The pre-refeeding phosphate concentration of 0.63 mmol/L supported cautious energy advancement, thiamine administration, and electrolyte replacement (21). The magnitude of weight and functional recovery shows that even extreme depletion may be reversible when nutrient delivery is restored and intestinal inflammation is at least partly controlled.

The diagnostic course also illustrates a common pitfall. Lymphocytic colitis in 2019 was a plausible and treatable explanation for persistent diarrhea, but treatment produced only partial improvement while nutritional decline continued. In non-responsive celiac disease, one alternative diagnosis should not end the investigation when response is incomplete or malnutrition progresses. Structured referral and early immunophenotypic assessment are recommended in this setting (22). Coexisting mycosis fungoides further complicated interpretation and appropriately prompted skin biopsy, although a single case cannot establish whether this association was coincidental or reflected shared immune dysregulation.

This case is relevant to North African and broader Mediterranean practice. Wheat-based foods are central to regional diets, making long-term gluten exclusion socially and economically demanding; expert dietetic verification is therefore a diagnostic prerequisite. At the same time, flow cytometry, clonality analysis, and capsule endoscopy remain unevenly available. When the aberrant population is unambiguous, immunohistochemistry using the >50% threshold can support RCD II, but absence of flow cytometry and clonality testing reduces diagnostic granularity and limits longitudinal quantification (5, 8).

The absence of flow cytometry and TCR clonality analysis is a substantive diagnostic limitation rather than a minor technical omission. Contemporary guidance recommends integrating immunohistochemistry, flow cytometry, and TCR rearrangement studies to distinguish RCD subtypes (5, 6). The >50% aberrant IEL phenotype by immunohistochemistry strongly supported RCD II in this patient, but it could not fully substitute for multimodal confirmation or provide the same precision for longitudinal quantification. The diagnosis should therefore be interpreted as the best-supported working classification based on the available tests, with repeat expert pathological assessment if additional tissue or molecular testing becomes available.

Therapeutic options beyond corticosteroids remain unsatisfactory. Immunosuppressive drugs may improve symptoms without reliably preventing EATL, while cladribine, autologous stem-cell transplantation, and therapies targeting interleukin-15 or JAK–STAT signaling have variable or still-evolving evidence (6, 7, 23, 24). In this patient, chronic hepatitis B, mycosis fungoides, and previous hemophagocytic lymphohistiocytosis made avoidance of prolonged systemic corticosteroid exposure particularly relevant.

### 3.1 Strengths and limitations

The strengths of this case are its multidisciplinary management and paired clinical, nutritional, biochemical, endoscopic, and immunohistochemical follow-up, which document the divergence between nutritional recovery and intestinal disease activity using objective longitudinal measures. The principal limitations are the single-patient design and absence of a comparator; the temporal association between treatment and improvement cannot establish efficacy or causality and cannot be generalized. The lack of flow cytometry and TCR clonality analysis is a major diagnostic limitation because RCD II was not confirmed by the full recommended multimodal approach. Nutritional and pharmacological interventions were also administered sequentially or concomitantly, preventing isolation of the independent effect of budesonide. Other limitations are the short 6-month follow-up; the 4-month timing of repeat biopsy; unavailable capsule endoscopy and HLA typing; incomplete documentation of the enteral access device, precise open-capsule manipulation, rationale for the initial 6-mg dose, antibiotic regimen, parenteral formulation, and exact energy-escalation schedule; unavailable exact follow-up values for several laboratory and anthropometric measures; and absence of validated symptom and quality-of-life instruments. Histological and immunohistochemical photographs of sufficient publication quality from both time points were not available from the Department of Pathology, preventing a reliable visual before-and-after presentation. Accordingly, the term nutritional remission is descriptive and must not be interpreted as mucosal healing, immunophenotypic remission, or eradication of RCD II. These limitations require cautious interpretation and long-term specialist surveillance.

### 3.2 Take-away lessons

Progressive malnutrition despite dietitian-verified adherence to a GFD should prompt early evaluation for refractory disease and EATL. Severe RCD II requires protocol-driven nutritional rehabilitation, selection of the feeding route according to intestinal function, and explicit prevention of refeeding syndrome. Most importantly, clinical and nutritional remission should not be interpreted as eradication of RCD II; endoscopic and immunophenotypic surveillance must continue independently of visible recovery.

## 4 Patient perspective

The patient reported that, during the years preceding the diagnosis, she felt increasingly disbelieved because persistent symptoms were repeatedly attributed to dietary non-adherence despite her efforts to follow the GFD. She described loss of independence as the most distressing aspect of her illness and recalled arriving at the hospital convinced that she might not survive. Learning that refractory disease, rather than personal failure, explained the deterioration increased her willingness to engage with treatment. She found parenteral nutrition difficult but accepted it after its temporary role had been explained. Regaining the ability to walk and prepare meals marked recovery for her. She was disappointed when told that the intestinal biopsies remained abnormal but preferred to know and understood the need for continued surveillance. She consented to publication in the hope that other patients might avoid a similar diagnostic delay.

## Data Availability

The original contributions presented in the study are included in the article/supplementary material, further inquiries can be directed to the corresponding author.
